# Ensembles of randomized trees using diverse distributed representations of clinical events

**DOI:** 10.1186/s12911-016-0309-0

**Published:** 2016-07-21

**Authors:** Aron Henriksson, Jing Zhao, Hercules Dalianis, Henrik Boström

**Affiliations:** Department of Computer and Systems Sciences, Stockholm University, Borgarfjordsgatan 12, Kista, SE-16407 Sweden

**Keywords:** Random forest, Distributional semantics, Heterogeneous data, Electronic health records, Pharmacovigilance, Adverse drug events

## Abstract

**Background:**

Learning deep representations of clinical events based on their distributions in electronic health records has been shown to allow for subsequent training of higher-performing predictive models compared to the use of shallow, count-based representations. The predictive performance may be further improved by utilizing multiple representations of the same events, which can be obtained by, for instance, manipulating the representation learning procedure. The question, however, remains how to make best use of a set of diverse representations of clinical events – modeled in an ensemble of semantic spaces – for the purpose of predictive modeling.

**Methods:**

Three different ways of exploiting a set of (ten) distributed representations of four types of clinical events – diagnosis codes, drug codes, measurements, and words in clinical notes – are investigated in a series of experiments using ensembles of randomized trees. Here, the semantic space ensembles are obtained by varying the context window size in the representation learning procedure. The proposed method trains a forest wherein each tree is built from a bootstrap replicate of the training set whose entire original feature set is represented in a randomly selected set of semantic spaces – corresponding to the considered data types – of a given context window size.

**Results:**

The proposed method significantly outperforms concatenating the multiple representations of the bagged dataset; it also significantly outperforms representing, for each decision tree, only a subset of the features in a randomly selected set of semantic spaces. A follow-up analysis indicates that the proposed method exhibits less diversity while significantly improving average tree performance. It is also shown that the size of the semantic space ensemble has a significant impact on predictive performance and that performance tends to improve as the size increases.

**Conclusions:**

The strategy for utilizing a set of diverse distributed representations of clinical events when constructing ensembles of randomized trees has a significant impact on predictive performance. The most successful strategy – significantly outperforming the considered alternatives – involves randomly sampling distributed representations of the clinical events when building each decision tree in the forest.

## Background

The digitization of healthcare in electronic health record (EHR) systems has opened up the possibility for analyzing the inexorably growing amounts of healthcare data with computational methods. Meaningful secondary use of healthcare data has the potential to make healthcare more effective and more efficient. Healthcare has indeed become an important application domain for machine learning and natural language processing; however, this valuable data source remains largely untapped [1]. One promising use of the vast amounts of healthcare data is for training predictive models that can support clinicians. There are, however, numerous challenges involved in learning high-performing predictive models from healthcare data. The high-dimensional nature of the data, as a consequence of the large amount of clinical events that can be used to describe an instance (e.g., a patient or a care episode), presents one such challenge. The high dimensionality of the data, in turn, typically renders it extremely sparse since patients, particularly within a given care episode, are only exposed to a very small subset of the clinical events used for describing the training sample. This is known as the *curse of dimensionality* and makes it difficult to apply statistical methods to healthcare data. Another challenge is presented by the inherent heterogeneity of such data, which entails that clinical events of different data types cannot be effectively handled in a uniform manner. A simplifying yet useful distinction is sometimes made between structured and unstructured data. Structured EHR data includes diagnosis codes (in the form of, e.g., ICD), drug codes (in the form of, e.g., ATC) and measurements (typically in the form of institution-specific encoding). Using these data types inevitably gives rise to questions of representation, how to handle values missing at random or not, and how to take into account the temporality of clinical events. These issues have been addressed in a number of studies [2–7].

The unstructured or semi-structured EHR data comes primarily in the form of free-text clinical notes written or dictated by clinicians. This data type – even more high-dimensional and sparse than the aforementioned structured data types – is particularly challenging to analyze computationally in this domain since it tends to be very noisy [8]: clinical notes often comprise telegraphic sentences that do not comply with formal grammar and contain numerous (non-standard) abbreviations and acronyms that are sometimes difficult to disambiguate even in context [9]. Here, too, the question of representation naturally presents itself and various alternatives have been explored [10].

We have previously proposed a means of representing heterogeneous data types by first learning deep representations of clinical events based on their distribution in EHRs. These representations are obtained by leveraging distributional semantics, i.e., techniques conventionally used in natural language processing for obtaining vector representations of words based on word co-occurrence information. The theoretical foundation underpinning models of distributional semantics is the distributional hypothesis [11], according to which words with similar meanings tend to appear in similar contexts, i.e., co-occur with largely overlapping sets of words. Distributed representations of words have been successfully exploited in a range of downstream natural language processing applications [12], also in the biomedical [13] and clinical [14] domains. In the clinical domain, the use of distributed representations has been investigated for applications such as medical terminology construction [15–18], medical concept recognition [19, 20], semi-automatic diagnosis coding [21–23], care episode retrieval [24], and detection of adverse drug events [10, 25–28]. In one of these studies [27], the distributional semantics framework was extended to other, non-linguistic but sequential, data types, which allowed distributed representations to be learned for diagnosis codes, drug codes and clinical measurements, in addition to words used in clinical notes. It was shown, in that study, that using these deeper representations of clinical events led to the construction of higher-performing predictive models compared to the use of more shallow representations, whereby each example was treated as a bag of clinical events. Modeling EHR data in so-called semantic spaces has three distinct advantages:

1. It mitigates the twin problems of high dimensionality and sparsity by creating dense, reduced-dimensional representations of the data. The dimensionality is controlled by the dimensionality of the distributed vectors representations, which is a model hyperparameter, effectively making the method scalable since the dimensionality does not grow with the size of the data.
2. It takes into account and explicitly models similarities between clinical events instead of treating them as atomic units about which we presumably know nothing a priori. The assumption here is that clinical events that have similar distributions in EHRs are, in some sense, semantically similar.
3. The representation learning procedure is unsupervised and thereby allows large amounts of unlabeled data, which tend to more readily available, to be leveraged.

This way of representing EHR data also makes it more feasible to combine clinical events of heterogeneous data types: the study showed that combining structured and unstructured EHR data led to significant improvements in predictive performance. Another finding of that study was that modelling each type of clinical event in a separate semantic space and then concatenating their representations was more effective than having a shared semantic space for the three structured data types.

In another study [28], it was demonstrated that the predictive performance could be further improved by leveraging multiple – and to some degree diverse – representations of the same events. The motivation behind this idea is to capture multiple, distributed views of the data in an *ensemble of semantic spaces* [29]. This notion has been explored for a number of applications, including medical terminology construction [18, 30], medical concept recognition [20, 31] and adverse drug event detection [28]. The multiple representations can be obtained by, for instance, manipulating the representation learning procedure. One means of creating a semantic space ensemble – studied previously and also used in the present study – is to vary the context definition, i.e., the region in which co-occurrences are considered, typically a window of surrounding items. This has been shown to affect the semantic properties that are modeled [32–34]. Once a set of potentially diverse semantic spaces has been created, the question arises of how to combine them in an effective manner. In the aforementioned study, early (feature) and various late (classifier) fusion strategies were investigated and it was shown that early fusion outperformed the considered late fusion strategies. In the present study, we investigate alternative ways of making use of semantic space ensembles in conjunction with ensemble methods bagging and random subspacing used in the random forest learning algorithm.

The random forest learning algorithm [35] creates an ensemble of decision trees that collectively vote, typically through some form of (weighted) majority voting, for which class label to assign to an instance. For an ensemble like this to be effective – that is, obtain a higher predictive performance than the individual, base models of which it is composed – its base models need to be accurate and diverse [36]. An explanation for the effectiveness of ensemble models can be traced back to the 18th century and Condorcet’s jury theorem [37], which states that the error of the majority of a jury decreases with the number of jury members. This theorem holds under the assumption that each member is more likely to be correct than wrong (i.e., accurate), but also requires that the members make the errors independently (i.e., are diverse [38]). The latter means, for example, that nothing is gained from forming a jury whose members always agree; the overall error will be no lower than the error of its members. Along with the number of base models, these two components – the performance of each base model and the extent to which the models vary in their predictions – affect the predictive performance of the ensemble. The random forest algorithm attempts to create diverse base models in two ways: (1) by training each decision tree from a bootstrap replicate of the original training set of *D* examples, i.e., sampling *D* examples with replacement from the training set (i.e., bagging); and (2) only allowing a random subset of the original features to be inspected when deciding on a splitting criterion at each node in the tree (i.e., random subspacing). An important question for ensemble models like random forest is how, precisely, the accuracy of the base models and their diversity relate to ensemble performance. In a regression framework, i.e., when the task is numerical prediction, the (squared) error E of the ensemble is directly related to the average (squared) error A of the base models, and their diversity D, i.e., the average (squared) deviation of each single prediction from the ensemble prediction (Eq. 1) [39].

1 $$ E = A - D  $$

The above states that the ensemble error can be no higher than the average base model error, and that the more diversity there is, the lower the ensemble error will be. It should, however, be noted that using the above directly in the search for an optimal ensemble is not straightforward, as there is normally a strong interplay between diversity and average base model performance, e.g., perfect base models will agree on all predictions. When it comes to classification accuracy, there is no similarly direct decomposition of ensemble performance into average base model accuracy and diversity. A large number of alternative diversity measures have been proposed in the literature [38]; however, their connection to ensemble performance have been shown to be questionable.

In this study, forests of trees that are trained from diverse distributed representations of clinical events, sampled at random, are evaluated for their ability to detect the presence of a particular adverse drug event (ADE) in care episodes documented in EHRs. Adverse drug events – defined as undesired harm resulting from the use of a drug – are the most common form of iatrogenic injury, causing approximately 3.7 % of hospital admissions worldwide [40]. Electronic health records have emerged as a potentially valuable source for pharmacovigilance, which, due to the limitations of clinical trials in terms of duration and sample size, needs to be carried out throughout the life-cycle of a drug to inform decisions about its continued use in the treatment of patients. A challenge for pharmacovigilance is that ADEs are heavily underreported [41], both in spontaneous reporting systems – to which ADE case reports are submitted voluntarily by patients and clinicians – and in EHRs, wherein ADEs can be encoded by a limited set of diagnosis codes. To address the problem of underreporting, alerting systems that can automatically detect ADEs in EHRs are potentially very valuable.

## Methods

This paper investigates three different strategies for utilizing a set of semantic spaces that contain distributed representations of the same clinical events. The investigation is carried out on 27 real, clinical datasets that are used for learning the binary classification task of detecting care episodes in which the patient has experienced a particular ADE. A number of follow-up experiments are then conducted in an attempt to identify possible sources for the observed differences in predictive performance, as well as to study the impact of the size of the semantic space ensemble on predictive performance.

### Modeling heterogeneous clinical events in semantic space

To create deep representations of clinical events, the data first needs to be presented as a sequence. For each of the three structured data types, we extract all sequences of events that occur in the healthcare episodes of patients, ordered by time. These sequences are then processed one-by-one by the distributional semantics algorithm. For notes, we obtain sequences of words. The preprocessed notes – lemmatized, without digits and punctuation – are processed sentence-by-sentence.

In this study, word2vec [42] is used to construct semantic spaces from the sequential data. This implements a recently developed model that has been inspired by research in deep learning and neural network-based language models. It was chosen for its ability to produce high-quality vector representations of words, outperforming traditional context-counting based methods on a range of natural language processing tasks [43] and now considered state-of-the-art in distributional semantics. We employ the skip-gram architecture. The algorithm constructs a vocabulary from the training data and learns vector representations of the sequential items (here, clinical events). It achieves this by training a neural network with a single hidden layer; given a set *D* of sequential items *i* and their contexts *c*, the objective function is to set the parameters *Θ* that maximize *p*(*c*|*i*;*Θ*) [44]:

2 $$  \mathop{\arg\,\max}\limits_{\Theta} \prod_{(i,c) \in D} p(c|i;\Theta)  $$

Context is defined as an adjacent item within a (symmetric) window of a pre-specified size around the input item. The parameters that are learned in the hidden layer give us the semantic vectors.

A semantic space is then created for each pre-specified context window size and set of input sequences. There is one set of input sequences for each data type: words, drug codes, diagnosis codes (for diagnosis codes, 27 variants are created wherein the target ADE label code is excluded to avoid bias) and measurements. In this study, the question is how best to utilize the set of diverse representations of clinical events that have been learned.

### Semantic space ensemble utilization strategies

The following three semantic space ensemble utilization strategies are investigated in this study. The first is essentially the baseline and corresponds to the early (feature) fusion approach with which the best results were obtained in a previous study [28]. Two variants of an alternative approach, wherein diverse distributed representations are sampled at random, are compared to the feature fusion approach. In all strategies, the distributed representations from each semantic space are treated as a bag, in the sense that the vector corresponding to a given clinical event is multiplied by its count – the number of times it has occurred in a given example – before being added to the vector corresponding to a given semantic space for that example. Distributed representations from semantic spaces generated with the same context window size but comprising different data types are, in all strategies, concatenated. However, the manner in which distributed representations from different types of semantic spaces, i.e. ones that have been generated with different context window sizes (henceforth referred to as different *types* of semantic spaces/distributed representations), differs in the three strategies. All strategies use bagging, two in combination with random subspacing, to create randomized trees. The combination of bagging and random subspacing has been shown to yield comparable performance to random forest proper, with the advantage of being applicable to any base classifier [45]. The utilization strategies are described in more detail below:

- **Fused Diverse Representations (FDR)**: The multiple distributed representations of the clinical events in the dataset are first concatenated; each tree in the forest is then generated from a bootstrap replicate of the transformed dataset and a random subset of the transformed features.
- **Randomized Diverse Representations with Feature Subsampling (RDR-FS)**: A single type of distributed representation is randomly selected for each tree, which is generated from a bootstrap replicate of the transformed dataset and a random subset of the transformed features.
- **Randomized Diverse Representations without Feature Subsampling (RDR-ALL)**: A single type of distributed representation is randomly selected for each tree, which is generated from a bootstrap replicate of the transformed dataset; however, in contrast to the previous strategy, the entire transformed feature set is used for building each tree.

The RDR-ALL strategy is also described in Algorithm 1. The only difference between RDR-FS and RDR-ALL is that the former makes use of random subspacing, while the latter does not but instead allows each tree to exploit the entire feature set. In this study, the distributed vectors are 200-dimensional; four data types are considered; ten types of semantic spaces (i.e., ten context window sizes are used;) and $\sqrt {N}$ features are randomly sampled when using feature subsampling. The number of features post transformation for each utilization strategy with this particular setup is shown in Table 1. If *V* is the vector dimensionality, *T* the number of data types, *P* number of types of semantic spaces (in this case the number of context window sizes) and $\sqrt {N}$ is chosen for feature subsampling, all three strategies allow the ensemble to exploit *V*×*T*×*P* features. For each decision tree, FDR allows $\sqrt {V \times T \times P}$ features to be exploited; RDR-FS allows $\sqrt {V \times T}$ features to be exploited; RDR-ALL allows *V*×*T* features to be exploited. The number of features is independent from the dimensionality of the original dataset.

**Table 1:** The number of features available to the ensemble and each tree with the three utilization strategies

Utilization strategy	Ensemble features	Tree features
FDR	8000	$\sqrt {8000}$
RDR-FS	8000	$\sqrt {800}$
RDR-ALL	8000	800



### Data source

The 27 datasets used in the following experiments were extracted from a subset of the Stockholm EPR Corpus [46]. This subset contains health records written in Swedish of around 700,000 patients over a two-year period (2009–2010) from Karolinska University Hospital in Stockholm, Sweden. This research has been approved by the Regional Ethical Review Board in Stockholm (permission number 2012/834-31/5).

The semantic space ensemble utilization strategies are here evaluated in the context of ADE detection. More precisely, the learning task is to detect care episodes that involve a certain ADE, i.e., care episodes in which an ADE-specific ICD-10 diagnosis code has been assigned. A care episode is here defined based on the time interval between recorded activities for a patient: a care episode is delimited by at least three days of no registered activities. The care episodes are described by four types of data: clinical notes, ICD-10 diagnosis codes, ATC drug codes and clinical measurements (represented as types, i.e., values are ignored). Only care episodes that contained at least one of each of the four data types were retained. Each of the 27 datasets thus consists of care episodes according to the above definition, where the positive examples have been assigned an ADE-related diagnosis code, i.e., have experienced a drug-induced disorder, and the negative examples are an equal number of randomly selected care episodes in which that same code has not been assigned. The ADE-related diagnoses were selected on the basis of having been classified as indicating ADEs in a previous study [47] and being sufficiently frequent (> 10 care episodes) in the used subset of the Stockholm EPR Corpus. The number of visits and characteristics of the datasets are described in Table 2. In addition to the labeled datasets, the entire two years of data in the subset is used for building the semantic spaces. That is, this is the dataset from which the distributed representations in the experiments are sampled. The notes are preprocessed by using Stagger [48] for tokenization and lemmatization of Swedish text and by removing all digits and punctuation. The notes contain approximately 3M unique words (700 M instances), while there are 9,046 diagnosis codes (51.6 M instances), 1,272 drug codes (2.9 M instances) and 713 measurements (14.5 M instances).

**Table 2:** Description of datasets

				Words (Lemmas)			Diagnoses (ICD-10)			Drugs (ATC)			Measurements	
Dataset		Visits		Types	Instances		Types	Instances		Types	Instances		Types	Instances
D64.2		416		46125	2110354		536	6320		364	8960		304	60689
E27.3		34		9564	112789		143	248		157	662		138	3982
F11.0		76		12200	232203		180	367		159	687		157	3920
F11.2		308		30077	904496		486	1875		347	4329		260	23637
F13.0		120		14764	215626		232	390		204	1167		153	6178
F13.2		76		12507	215321		220	484		195	922		167	4621
F15.0		32		5849	39658		71	148		96	257		105	1427
F15.1		46		9174	102697		122	259		142	573		137	4518
F15.2		256		25179	658428		394	1347		295	3439		209	22870
F19.0		122		15823	278873		237	475		214	1120		227	5519
F19.1		74		12651	177644		186	373		186	985		152	4688
F19.2		288		29291	799717		492	1259		326	3667		262	19653
F19.9		68		13144	177749		177	350		178	992		87	3743
G24.0		28		10017	101769		76	132		136	599		113	3551
G62.0		20		4622	35997		41	71		93	219		56	1119
I95.2		70		11528	145432		162	652		177	799		144	5252
L27.0		274		34504	1114979		556	1619		375	5324		273	28451
L27.1		78		13477	234268		220	545		186	1260		128	6088
N14.1		28		9180	82075		105	387		128	335		99	2215
O35.5		128		10567	121849		278	882		223	1654		125	3894
T59.9		40		5803	47694		81	165		104	317		76	1467
T78.2		102		13341	188250		208	602		200	1063		200	5384
T78.3		266		22659	411014		393	1178		282	2454		208	9967
T78.4		1520		46575	1633049		926	4571		463	9567		370	39883
T80.8		732		39077	1655988		709	5323		425	9890		269	35283
T88.6		96		15137	227317		240	549		209	1290		185	6325
T88.7		564		42794	1436333		767	3303		467	7263		306	41793

### Experimental setup

As mentioned previously, word2vec and the skig-gram model is used for generating semantic spaces with 200-dimensional vectors. The following ten context window sizes are used: 2+2, 4+4, 6+6, 8+8, 10+10, 12+12, 14+14, 16+16, 18+18, 20+20. With four types of clinical events – words, diagnosis codes, drug codes and measurements – this results in 40 semantic spaces; however, in reality, there are even more semantic spaces since a separate semantic space is generated for each window size and diagnosis code, where the target diagnosis code has been excluded to avoid bias.

Forests are built with 500 trees and, when random subspacing is employed, i.e., for FDR and RDR-FS, each tree is able to exploit $\sqrt {N}$ features randomly sampled from the original feature set of size *N*. Predictive performance is estimated using 10-fold cross validation, save for in one of the follow-up experiments were randomized train-test splits are used. The considered performance metrics are accuracy and area under the ROC curve (AUC). Accuracy corresponds to the percentage of correctly classified instances, while AUC estimates the probability that a model ranks a randomly chosen positive instance ahead of a negative one. A Friedman test, followed by a post-hoc test using the Bergmann-Hommel procedure, as suggested in [49], is employed for statistical hypothesis testing, where the null hypothesis is that the methods perform equally well; the ranks are compared, adjusting for the fact that multiple comparisons are made.

Three experiments are conducted in this study. In the first and main experiment, the three semantic space ensemble utilization strategies are evaluated w.r.t. accuracy and AUC using 10-fold cross-validation over the 27 ADE datasets. A Friedman test is applied to assess whether the strategies have a significant impact on predictive performance, followed by a post-hoc test to assess the significance of pairwise differences. The second experiment – and the first of two follow-up analyses – involves inspection of the ensemble models in an attempt to uncover the source of differences in predictive performance. To that end, we look at average tree accuracy and diversity, measured as ensemble accuracy minus average tree performance. Again, this is estimated using 10-fold cross-validation, while a Friedman test, followed by a post-hoc test, is applied to assess whether the observed differences are statistically significant. The third and final experiment constitutes another follow-up analysis, in which the best-performing strategy is employed when assessing the impact of the semantic space ensemble size on predictive performance. The considered sizes for the pool of window sizes are: 1, 2,..., 10. All possible combinations of semantic spaces to include from the original pool are evaluated using randomized 70–30 train-test splits and averaged. A Friedman test is used to asses whether pool size, i.e., the number of semantic spaces included in the ensemble, has a significant impact on predictive performance.

## Results

The evaluation of the three utilization strategies on the 27 ADE datasets shows that the RDR-ALL strategy yields the highest predictive performance w.r.t both accuracy and AUC, while RDR-FS leads to the worst performance (Table 3). The differences among the three strategies are statistically significant for accuracy (=0.0023) but not for AUC. A post-hoc test shows that RDR-ALL leads to significantly higher accuracy than FDR (*p*=0.04122) and RDR-FS (*p*=0.00156). There is, however, no pairwise significant difference between FDR and RDR-FS. For the sake of reference, all of the results are substantially higher than those obtained with a shallow, count-based representation instead of diverse distributed representations: this yields, on average, 84.11 % accuracy and 0.923 AUC.

**Table 3:** Predictive performance with the three strategies over 27 datasets

Dataset	Accuracy % (Rank)			AUC (Rank)		
	FDR	RDR-FS	RDR-ALL	FDR	RDR-FS	RDR-ALL
D642	95.19 (1.5)	93.76 (3.0)	95.19 (1.5)	0.974 (1.0)	0.967 (3.0)	0.969 (2.0)
E273	77.50 (3.0)	80.00 (2.0)	85.00 (1.0)	0.923 (2.0)	0.954 (1.0)	0.902 (3.0)
F110	91.25 (3.0)	92.92 (1.5)	92.92 (1.5)	0.956 (3.0)	0.966 (1.0)	0.958 (2.0)
F112	88.27 (3.0)	88.98 (2.0)	90.27 (1.0)	0.950 (2.0)	0.937 (3.0)	0.960 (1.0)
F130	90.83 (2.0)	90.83 (2.0)	90.83 (2.0)	0.958 (2.0)	0.952 (3.0)	0.961 (1.0)
F132	89.58 (1.5)	86.67 (3.0)	89.58 (1.5)	0.938 (2.0)	0.930 (3.0)	0.977 (1.0)
F150	90.00 (2.0)	90.00 (2.0)	90.00 (2.0)	0.876 (2.0)	0.875 (3.0)	0.876 (1.0)
F151	89.17 (2.0)	90.00 (1.0)	85.00 (3.0)	0.990 (1.0)	0.954 (3.0)	0.990 (2.0)
F152	94.97 (2.0)	94.58 (3.0)	95.32 (1.0)	0.979 (1.0)	0.978 (2.0)	0.977 (3.0)
F190	90.83 (2.0)	90.00 (3.0)	90.95 (1.0)	0.958 (2.0)	0.960 (1.0)	0.957 (3.0)
F191	87.50 (2.0)	83.75 (3.0)	88.75 (1.0)	0.961 (3.0)	0.962 (2.0)	0.977 (1.0)
F192	90.21 (1.0)	89.90 (3.0)	90.19 (2.0)	0.942 (3.0)	0.956 (1.0)	0.947 (2.0)
F199	87.08 (2.0)	82.92 (3.0)	88.75 (1.0)	0.959 (1.0)	0.939 (3.0)	0.955 (2.0)
G240	87.50 (3.0)	90.00 (2.0)	92.50 (1.0)	1.000 (1.0)	0.924 (3.0)	0.973 (2.0)
G620	90.00 (1.5)	85.00 (2.0)	90.00 (1.5)	0.900 (2.0)	0.900 (2.0)	0.900 (2.0)
I952	87.50 (2.0)	85.00 (3.0)	88.75 (1.0)	0.932 (2.0)	0.883 (3.0)	0.956 (1.0)
L270	85.05 (1.0)	83.60 (3.0)	84.31 (2.0)	0.917 (2.0)	0.917 (3.0)	0.920 (1.0)
L271	73.33 (3.0)	74.58 (2.0)	78.33 (1.0)	0.798 (3.0)	0.804 (1.0)	0.799 (2.0)
N141	70.00 (2.0)	67.50 (3.0)	72.50 (1.0)	0.800 (3.0)	0.830 (1.0)	0.825 (2.0)
O355	99.17 (2.5)	99.17 (2.5)	100.0 (1.0)	1.000 (2.0)	1.000 (2.0)	1.000 (2.0)
T599	92.50 (2.5)	97.50 (1.0)	92.50 (2.5)	1.000 (2.0)	1.000 (2.0)	1.000 (2.0)
T782	84.17 (3.0)	85.17 (2.0)	88.17 (1.0)	0.925 (2.0)	0.924 (3.0)	0.931 (1.0)
T783	90.00 (2.0)	88.16 (3.0)	90.36 (1.0)	0.951 (2.0)	0.946 (3.0)	0.955 (1.0)
T784	93.16 (2.0)	92.17 (3.0)	93.68 (1.0)	0.981 (2.0)	0.982 (1.0)	0.980 (3.0)
T808	94.93 (1.0)	93.97 (3.0)	94.92 (2.0)	0.982 (1.0)	0.979 (3.0)	0.982 (2.0)
T886	84.50 (2.0)	84.50 (2.0)	84.50 (2.0)	0.914 (1.0)	0.882 (3.0)	0.910 (2.0)
T887	84.03 (1.0)	83.14 (2.0)	82.62 (3.0)	0.896 (1.0)	0.892 (2.0)	0.890 (3.0)
Mean	88.08 (2.1)	87.55 (2.4)	89.11 (1.5)	0.939 (1.9)	0.933 (2.3)	0.942 (1.9)
*p*-value	0.0023			0.2540		

**Fig. 1:**
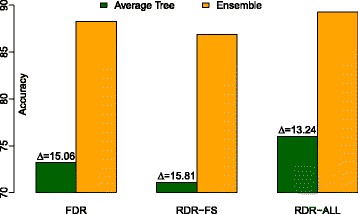
Ensemble inspection: average tree and ensemble performance with the three strategies. The deltas indicate the amount of diversity in the ensembles

A follow-up experiment was conducted to investigate what the differences in predictive performance stem from. An attempt to that end was made by looking into the ensemble models produced with the three strategies and estimating average tree accuracy versus ensemble accuracy. The results of these experiments are depicted in Fig. 1. The accuracy scores of the ensembles have already been presented; what is new is instead the average tree accuracy scores. We can see that RDR-ALL obtains not only the highest ensemble performance but also the highest average tree performance, while RDR-FS similarly obtains the lowest average tree performance. Diversity is here crudely estimated as the difference between ensemble performance and average tree performance; we can observe that RDR-ALL seems to exhibit the least amount of diversity. A Friedman test shows (Table 4) that the three strategies have a statistically significant impact on average tree accuracy (*p*<0.0001) and diversity (*p*<0.0001). In fact, RDR-ALL obtains the highest average tree accuracy on all 27 datasets. A post-hoc test moreover reveals (Table 5) that the pairwise differences in average tree performance are statistically significant (*p*<0.0001). The differences in diversity is statistically significant between all pairs save between FDR and RDR-FS.

**Table 4:** Ensemble inspection: average tree accuracy and diversity with the three strategies

Strategy	Average tree accuracy			Diversity		
	Mean score	Mean rank	*P*-value	Mean score	Mean rank	*P*-value
FDR	73.22	2.0	<0.0001	0.15	1.9	<0.0001
RDR-FS	70.99	3.0		0.16	1.6	
RDR-ALL	76.02	1.0		0.13	2.6

**Table 5:** *P*-values of pairwise differences between the three strategies w.r.t average tree accuracy and diversity

	Average tree accuracy	Diversity
FDR vs. RDR-FS	0.00007	0.27630
FDR vs. RDR-ALL	0.00001	0.00650
RDR-FS vs. RDR-ALL	<0.0001	0.00004

**Fig. 2:**
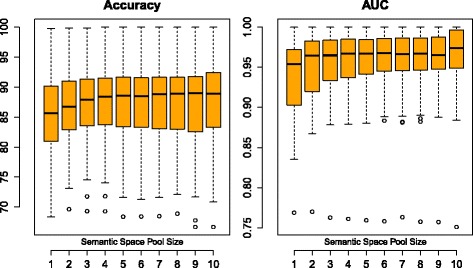
Predictive performance as the size of the semantic space ensemble is varied

Another follow-up experiment was conducted to investigate whether the size of the semantic space ensemble – that is, the number of diverse distributed representations to sample from – has an impact on predictive performance. The results of this analysis are depicted in Fig. 2, showing how the predictive performance is affected as the semantic space pool size – the number of types of semantic spaces that are included in the ensemble – is varied from 1 to 10 with a step-size of 1. As the boxplots show, the predictive performance, w.r.t. both accuracy and AUC, tends to improve with the size of the semantic space ensemble, although not monotonically so. A Friedman test confirms (Table 6) that the pool sizes that are investigated in this study have a statistically significant impact on both accuracy and AUC (*p*<0.0001).

**Table 6:** Average performance with different semantic space ensemble sizes

Pool size	Accuracy			AUC		
	Mean score	Mean rank	*P*-value	Mean score	Mean rank	*P*-value
1	84.80	8.7	<0.0001	0.934	9.3	<0.0001
2	86.12	6.5		0.945	8.0	
3	86.31	5.6		0.949	7.0	
4	86.43	5.2		0.950	6.4	
5	86.45	4.9		0.951	5.7	
6	86.45	5.4		0.952	4.6	
7	86.43	4.7		0.953	3.7	
8	86.60	4.4		0.953	3.3	
9	86.27	5.1		0.954	3.1	
10	86.98	4.4		0.957	3.8	

## Discussion

Three strategies for utilizing a set of semantic spaces were explored in this study, of which two proposed to randomly sample diverse distributed representations when building each decision tree in the forest. The strategy wherein random subsampling was not employed (RDR-ALL) yielded the highest predictive performance and significantly outperformed both the variant of this strategy with random subsampling (RDR-FS) and the strategy wherein the distributed representations were simply concatenated and provided to the learning algorithm (FDR). This is a strong result given that the FDR strategy had previously outperformed numerous late fusion strategies, wherein a separate ensemble was trained for each context window size and subsequently combined in various ways [28].

It is interesting that the choice of whether to employ random subspacing with the RDR approach has such a substantial and significant impact on predictive performance. As the first follow-up experiment revealed, RDR-ALL yielded a significantly higher average tree performance at the expense of losing some diversity – in fact, a significant amount thereof. This can be explained by the fact that RDR-ALL exploits the entire feature set while RDR-FS is only allowed to exploit a small subset of the features: allowing each tree to have access to the entire feature set improves its predictive performance, but results in the trees varying less in their predictions, i.e., they become less diverse. In the RDR approach, diversity is sought in two ways: by building each tree from a bootstrap replicate of the original dataset and by representing this in a randomly selected type of distributed representation.

It would of course be possible not only to sample from a type of distributed representation as defined by the employed context window size, but also from, for instance, a given data type. This would, however, not allow the entire feature set to be exploited by each tree – a component that proved important to the success of the RDR-ALL strategy – as well as exclude the possibility for the learning algorithm to exploit interactions between data types. This is indeed a possible explanation for the relative ineffectiveness of the late fusion strategies explored in the previous study [28], as it did not allow for interactions to be exploited between different context window sizes. This potential limitation holds, however, also for the RDR approach.

In the FDR and RDR-FS strategies, the original features were transformed to their distributed counterparts prior to applying random subspacing. An alternative to this, which could be explored in future work, would be to apply random subspacing first and then conduct the feature transformation. This would allow the distributed representations to be kept in tact, while exploiting random subspacing as means for creating diversity.

Like in most ensemble models, the size of the ensemble had a significant impact on its performance. In this study, a limited pool of ten window sizes was experiment with and it was shown that performance tended to increase with the size of the pool to sample from. In comparison to the previous study [28], the trend is much more stable with RDR-ALL than with FDR, which was used in a similar analysis. This could possibly be the consequence of merely averaging over a larger number of results, as not all combinations of semantic spaces to include were evaluated then. In any case, this is a desirable property of an ensemble – that its performance is not too w.r.t. sensitive to the selected number of constituent models. A general rule of thumb when using random forest, for instance, is that the more trees, the better. This generally seems to be the case with the RDR-ALL utilization strategy. It would be interesting to observe if the trend were to continue with even larger pool sizes.

## Conclusions

A strategy for utilizing a set of diverse distributed representations of clinical events, - modeled in an ensemble of semantic spaces - in conjunction with ensemble techniques used in the random forest learning algorithm was proposed: it is based on the notion of randomly sampling a type of distributed representation for each tree in the forest. It was shown that, when employing this approach, allowing each tree to exploit the entire transformed, distributed feature set was more effective than applying random subspacing, which is used in the random forest learning algorithm. The proposed utilization strategy significantly outperformed an early feature fusion approach whereby the diverse distributed representations are simply concatenated. The improved predictive performance seems to stem from higher average tree performance rather than increased diversity. It was also shown that the proposed utilization strategy exhibits a desirable property of ensembles, namely that performance improves with the size of the ensemble.
